# Use of Hormone Blockers in Transgender Teenagers: A Scoping Review

**DOI:** 10.3390/nursrep14040299

**Published:** 2024-12-20

**Authors:** M. J. Herrera Jerez, M. E. Castro-Peraza, N. M. Delgado Morales, A. Arias Rodriguez

**Affiliations:** 1 Doctoral Program in Medical and Pharmaceutical Sciences, Development and Quality of Life, University of La Laguna, 38200 Santa Cruz de Tenerife, Spain; manueljhj@icloud.com; 2 University Hospital of the Canary Islands, Canary Islands Health Service, 38320 Santa Cruz de Tenerife, Spain; 3 Primary Care Management of Tenerife, Canary Island Health Service, 38200 Santa Cruz de Tenerife, Spain; nayradelmor@icloud.com; 4 Area of Preventive Medicine and Public Health, University of La Laguna, 38200 Santa Cruz de Tenerife, Spain; angarias@ull.edu.es

**Keywords:** hormone blockers, transgender teenagers, dysphoria, mental health, nurse, review

## Abstract

Hormone blockers are defined as substances that suppress the release of sex hormones, thus inhibiting the development of secondary sexual characteristics in teenagers. There is currently an increase in young people seeking healthcare services due to a mismatch between their birth-assigned gender and their perceived or self-identified gender. In early childhood, individuals are not usually affected by their physical appearance. Dysphoria may arise during the initial stages of adolescence and if the self-perceived gender does not align with the external appearance. This may have a negative impact on adolescents’ mental health. Could the use of hormone blockers have a positive effect on mental health? The primary objective of this review is to assess whether the use of hormone blockers can have positive effects on the mental health of transgender youth. The review also seeks to evaluate the usage of hormone blockers and the diagnosis of gender dysphoria. A literature search of scientific evidence was conducted across various databases—PUBMED, CUIDEN, ELSEVIER, COCHRANE, DIMENSIONS, SCIELO, PSYCINFO, and CINAHL—alongside a review of the latest publications in high-impact scientific journals and the gray literature. The following terms were used: <trans people>, <hormone blockers>, <depression>, <anxiety>, <teenagers>, <trans teens>, and <dysphoria>. A time period was specified, covering the last ten years (2014–2024). The initial search identified a total of 290 references, which were subsequently narrowed down to 5 studies, with 1 additional study retrieved through other methods. The literature findings are clear. They show that the use of hormone blockers in transgender adolescents can be beneficial, as a reduction in mental health issues was observed during and after their use. Mental healthcare in transgender teenagers is of crucial importance to their physical, psychological, social, and academic spheres. It is also very important for their families. Nurses must be aware of this knowledge to improve the care provided to these individuals and their families during the difficult time surrounding decisions about the use of hormone blockers.

## 1. Introduction

There is an increasing number of transgender and gender-diverse youth—individuals whose gender identity does not align with the sex assigned to them at birth—seeking gender-affirming healthcare [1,2]. Hormone blockers or gonadotropin-releasing hormone agonists (GnRHas) are substances that block pituitary receptors (GnRH receptors), thereby inhibiting the development of secondary sexual characteristics. They can be used in boys and girls experiencing early puberty, which begins between the ages of 8 and 9 for girls and between 9 and 10 for boys [3]. GnRH analogs do not cause permanent physical changes in the short to medium term, allowing adolescence to continue once the treatment is stopped [4,5].

According to the current literature, the prevalence of transsexuality in children and teens ranges from 1.3% to 3.6% among school-aged children [6]. However, since the implementation of the Dutch Protocol, there has been an increase in the number of adolescents seeking treatment [7]. The term “trans” is inclusive and encompasses individuals whose gender identity and/or gender expression differs from cultural expectations based on their sex assigned at birth [8]. This includes transsexuals, transgender individuals, non-binary individuals, those with fluid gender expression, and other gender variations. It is a heterogeneous group [9], highly stigmatized and misunderstood by both the general population and some healthcare professionals [6,10]. Various sources report that members of this group face multiple forms of violence [6,11]. They are at high risk of abuse and mistreatment by parents and caregivers [6], which affects their mental health, particularly during preadolescence. These individuals exhibit high levels of depression, anxiety, and substance abuse, with an increased risk of suicide and a high prevalence of severe psychological distress, further aggravated when facing hate or discrimination [11,12].

Gender dysphoria indicates the discomfort experienced by individuals whose gender identity differs from the sex they were assigned at birth. Dysphoria can be accompanied by multiple psychiatric conditions, including depression, anxiety, low self-esteem, social phobias, eating disorders, and, in severe cases, suicide attempts, self-harm, or the use of uncontrolled hormones [4,9,12]. The prevalence of depression among transgender and gender-diverse youth provides evidence that depression rates in this population are higher compared to their cisgender peers [9,13]. In a group of transgender university students, there was double the risk of psychiatric conditions, particularly depression and anxiety. Among trans women, gender-based violence and depressive symptoms are associated with higher substance use rates [14].

The annual prevalence of eating disorder diagnoses and the use of weight loss pills, vomiting, or laxative intake are higher among transgender students than in other groups, and they are lowest among cisgender heterosexual men [15]. Transgender students have higher rates of eating disorder diagnoses compared to cisgender heterosexual women [9]. The lifetime prevalence of suicidal ideation in Spain is 4.4% [16]. However, among trans individuals, suicidal ideation rates exceed 50% [16,17]. In the school context, Clark’s study in New Zealand demonstrated that transgender students had higher suicide attempt rates in the past twelve months compared to cisgender students (19.8% vs. 4.1%) and higher rates of self-harm (45.5% vs. 23.4%) [9]. In a community setting, Connolly et al. found that 18.8% of young transgender women reported moderate/high suicidal tendencies in the previous 30 days [9].

A critical phase for trans people arises with the appearance of secondary sexual characteristics of the birth-assigned sex, potentially leading to the aforementioned gender dysphoria [4]. Some studies have highlighted the elevated risks of self-harm and suicidal ideation, and/or suicidal attempts, in transgender children approaching puberty [6]. Adolescence and early adulthood are stages with the highest risk of suicidal behavior [16]. Among the trans community, suicidal ideation is more frequent during adolescence [16]. The use of hormone blockers can alleviate distress in trans minors, providing time to weigh different options and creating a “pause” in development [4,5,6,18,19,20], allowing for the best possible process of transitioning—the adaptation of an individual’s sexual characteristics to their desired sex [21].

Nursing plays a vital role in the interdisciplinary care of transgender youth, particularly in the administration and monitoring of hormone blockers. Nurses provide psychosocial support to patients and families, guide decision-making processes, and collaborate with other healthcare professionals to ensure holistic and evidence-based care [22].

We consider this review to be important for nursing. The role of nursing, with its own body of knowledge, has varied throughout history, and the expansion of knowledge has qualitatively and quantitatively improved our care. Indeed, since The Crimean War, Florence Nightingale’s knowledge of statistics had an impact on the decrease in the mortality rate of soldiers. Currently, the challenges of nursing have changed, but the contribution of best evidence is always present to allow the individual, their family, and society to make the best possible health decisions. Nurses can manage the use of hormone blockers and synthesize patient information for future analysis.

The main objective of this review is to explore the impact of hormone blockers on the mental health of transgender youth and how access to these treatments is influenced by the requirement of a diagnosis of gender dysphoria. By addressing these interrelated aspects, this review aims to provide a comprehensive understanding of the use and effects of hormone blockers in this population.

## 2. Materials and Methods

A review was conducted to explore the following integrated question: What is the impact of hormone blockers on the mental health of transgender youth, and how do diagnostic criteria, such as the requirement for a gender dysphoria diagnosis, influence access to these treatments? This scoping review sought to map the evidence related to these interconnected issues. This review also explored the role of nursing in the care of transgender youth receiving hormone blockers, with a focus on psychosocial support, education, and interdisciplinary collaboration.

The review was conducted following the Arksey and O’Malley framework. The Arksey and O’Malley framework was chosen because it allows for a broad exploration of complex and interconnected topics, such as the mental health effects of hormone blockers and the role of diagnostic criteria in their access.

### 2.1. Search Strategy

The search was conducted according to a five-step methodological framework, with additional support criteria from Arksey and O’Malley:

Stage 1: Identification of the research question;

Stage 2: Identification of relevant studies;

Stage 3: Selection of studies based on the inclusion criteria;

Stage 4: Data extraction and reporting;

Stage 5: Compilation, summary, and communication of results.

The first phase involved an exploratory search to assess the topic and provide context and updates. New descriptors were added based on this search, such as <depressions> and <anxiety>, which were used in subsequent bibliographic searches. Boolean operators such as AND, OR, and NOT were employed to combine search terms like ‘hormone blockers AND transgender teens’, ‘depression OR anxiety AND hormone therapy’, and ‘gender dysphoria AND puberty suppression’.

Searches were conducted on the following bibliographic databases: PUBMED, CUIDEN, ELSEVIER, COCHRANE, DIMENSIONS, SCIELO, PSYCINFO, and CINAHL.

### 2.2. Study Selection Criteria

Meta-analyses and cohort and descriptive studies were prioritized. Priority was given to quantitative studies, such as controlled trials and longitudinal studies, due to their ability to establish causality or temporal relationships.

#### 2.2.1. Inclusion Criteria

Quantitative, qualitative, or mixed-method studies published in scientific journals on the impact of hormone blockers on transgender teenagers were selected. Studies published in Spanish or English were considered, and the search period was from February to March 2024.

#### 2.2.2. Exclusion Criteria

Editorials, commentaries, book reviews, and abstracts were excluded. The search began with a selection of articles based on title, abstract, and keywords, conducted by one reviewer. As only English and Spanish studies were included, this might have limited the generalizability of the findings to other cultural or linguistic contexts.

### 2.3. Data Analysis

A data extraction form was used to determine what data to collect. Data from the studies were extracted using a structured data sheet. For each included article, the following information was collected: author and year of publication, title, geographical location, study design and objectives, and a summary of the results.

## 3. Results

**Figure 1:**
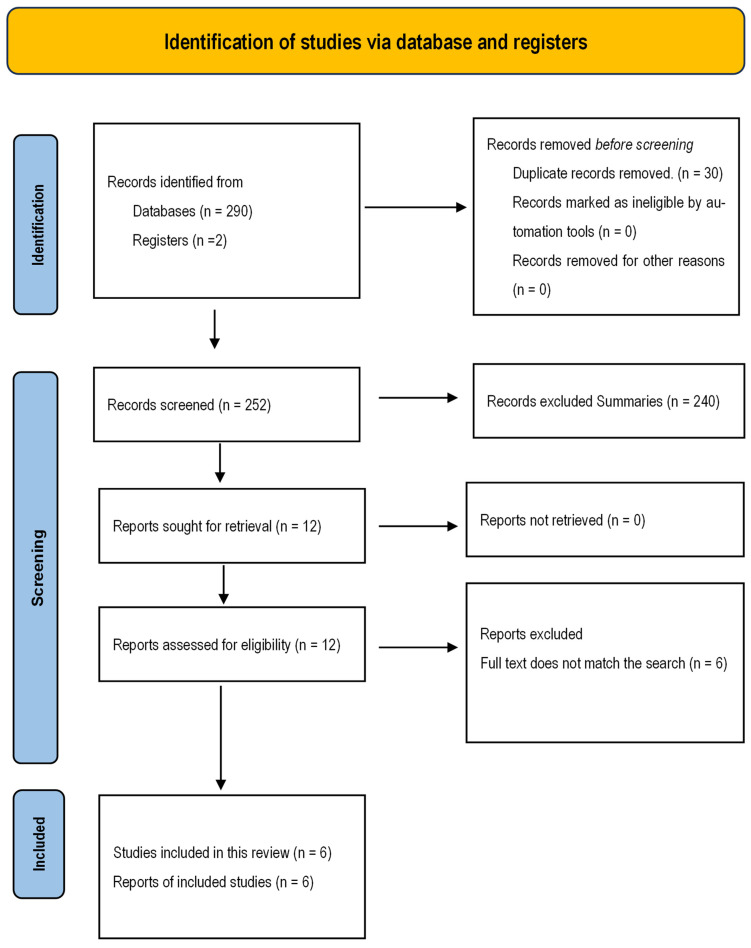
Flow diagram of the review process according to the PRISMA statement.

The initial search strategy identified 290 references across databases. After eliminating duplicates according to the publication’s objective, 250 references remained. They were screened by abstract, excluding 240 for not addressing the topic in question. A total of 10 studies were retrieved for full-text reading, and 5 were excluded because their full text did not align with the search objective. Thus, five studies were included, with one additional study identified through other methods, selected after applying the exclusion criteria. A total of six studies were included, and they are presented in the PRISMA flow diagram in Figure 1.

Table 1 presents the characteristics of the six studies included in this review. The studies are classified by author, year of publication, context, design and objective, participants, country where they were conducted, and key findings. Overall, this scoping review includes two systematic reviews [9,18], two longitudinal observational studies [6,19], and two retrospective cohort observational studies [7,23].

.

## 4. Discussion

This review highlights the fact that access to hormone blockers is influenced by diagnostic criteria, particularly the requirement for a gender dysphoria diagnosis. While the evidence supports positive mental health outcomes for transgender adolescents using hormone blockers, delays or barriers to treatment—often tied to diagnostic requirements—can exacerbate mental health challenges. This interconnection underscores the importance of considering both the clinical effects of these treatments and the criteria governing their accessibility. Thus, this review did not find conclusive data on the use of hormone blockers in transgender adolescents. However, the various studies included in this review support the existing evidence, revealing few severe adverse effects and several positive mental health outcomes for transgender adolescents, such as reductions in depression, anxiety, self-harm attempts, and suicidal ideation. Furthermore, improvements were noticed in quality of life and life satisfaction.

In this review, the various articles discuss the use of hormone blockers at Tanner Stage 2, and their effects on mental health and the diagnosis of gender dysphoria.

### 4.1. Use of Hormone Blockers

Most adolescents (93%) who use GnRHa later begin with gender-affirming hormone (GAH) therapy. This finding suggests that GnRHa treatment is used as an initiation of the transition process rather than an extension of the diagnostic phase [7]. Additionally, one study concluded that the use of GnRHa was not associated with an increased subsequent use of GH (growth hormone) [23].

### 4.2. Positive Effects of Hormone Blockers on Mental Health

With respect to the primary objective of this review, which was the use of hormone blockers in transgender adolescents, the various studies demonstrated an improvement in mental health outcomes, including psychological functioning (gender dysphoria, body image, global functioning, depression, anxiety, emotional and behavioral problems), objective well-being (social and educational/professional functioning), and subjective well-being (quality of life, life satisfaction, and happiness) [5,23]. Access to puberty suppression during adolescence was associated with a reduction in lifetime suicide rates among transgender adults [18].

Young people aged 9 to 25 who participated in gender-affirming endocrine treatment (i.e., puberty suppression or opposite-sex hormones) showed better mental health over time [18]. The use of GnRHa treatment in younger adolescents is reversible and associated with improvements in mental health and esthetic outcomes if gender dysphoria persists [22].

Regarding psychological functioning, significant improvements were found in general functioning alongside decreases in depressive symptoms and reductions in emotional and behavioral problems [18,23]. Notably, compared to youth who did not receive puberty suppression, those who did showed lower lifetime rates of suicidal ideation [18]. Access to puberty suppression during adolescence was associated with a reduction in lifetime suicide rates among transgender adults [18].

Regarding objective well-being, improvements in individuals’ affective and social lives were reported [18].

Regarding subjective well-being, a study conducted on adults exposed to the Dutch Protocol reported no regret among the participants during puberty suppression. Their quality of life, life satisfaction, and subjective happiness were comparable to those of their age-matched peers [5,7].

In a study which collected expert opinions on the use of hormone blockers in adolescents, the respondents confirmed that, after receiving family support, the majority agreed that puberty suppression reduced the traumatic pubertal changes they experienced due to severe bodily distress and social phobias. These experiences caused feelings of shame, self-loathing, guilt, potential self-harm, and one respondent continued to be affected 20 years later by an attempt to remove their genitalia [6].

### 4.3. Diagnosis of Gender Dysphoria

Gender dysphoria was present in most studies as a determining, though not entirely exclusive, factor for initiating the administration of hormone blockers [6,7,9,18,19]. The majority (71%) of the participants in these studies required a diagnosis of gender dysphoria to begin puberty suppression [18]. For example, a team of specialists in the Netherlands at the Amsterdam Gender Dysphoria Expertise Center observed that transgender individuals who underwent hormonal transition at younger ages more easily assimilated into their “new gender roles”, given that this diagnosis was present [7,9]. In fact, clinical observations described by Spack et al. suggested that the psychological functioning of these patients improved with medical intervention, implying that “psychiatric symptoms may be secondary to a medical incongruence between mind and body, rather than primarily psychiatric” [9].

In this review, no studies of meta-analytic techniques were found; furthermore, it was difficult to evaluate the different health services in the studies, which may be a limitation. The heterogeneity of the studies found may also be a limitation of the results. As stated before, the absence of meta-analytic techniques highlights the need for quantitative evidence to strengthen the conclusions. Cultural and healthcare system differences likely influence treatment applicability, suggesting the need for localized studies.

The findings of this review underscore the importance of nursing interventions in the care of transgender youth receiving hormone blockers. Nurses are integral to interdisciplinary teams, offering unique contributions such as guiding decision making, providing psychosocial support, and monitoring treatment outcomes. These roles are particularly critical in addressing mental health challenges and supporting families throughout the process.

## 5. Conclusions

The evidence regarding the administration of hormone blockers in transgender adolescents is very limited. The lack of longitudinal data remains a gap in the literature. Transgender children and adolescents are an understudied and poorly understood population.

Longitudinal cohort studies with conclusive data are needed to guide adolescents, families, and healthcare professionals in decision making, ensuring that the transition process is carried out in the best possible way and minimizing the negative physical and psychosocial outcomes that may be associated with pubertal development.

Multidisciplinary teams that guide transgender person in decision making are also made up of nurses. Given the little literature found on the use of hormone blockers in transgender teenagers, it is vitally important that we know about their action and that we provide the greatest possible evidence to our adolescents, being able to screen for any impact on their mental health.

Nursing interventions such as providing psychosocial support, guiding families in decision making, and collaborating with interdisciplinary teams are critical to improving care outcomes for transgender adolescents. Future research should prioritize patient-reported outcomes to capture subjective insights, such as quality of life and satisfaction with care. Additionally, studies should explore the specific contributions of nursing to the care of transgender adolescents, particularly in the domains of psychosocial support, education, and advocacy within interdisciplinary teams.

This review demonstrates that hormone blockers positively impact mental health outcomes in transgender adolescents, reducing depression, anxiety, and suicidal ideation. However, the requirement for a gender dysphoria diagnosis influences access to these treatments, potentially delaying care and exacerbating mental health challenges. Future research should address these interconnected issues to guide evidence-based policies and clinical practices. Longitudinal research and interdisciplinary collaboration are essential for advancing evidence-based care for this population.

## Figures and Tables

**Table 1:** Participants, design, and key findings of the studies included in this review.

Author and Year	Study Type	Objectives	Country	Participants and Context	Key Findings
Lynn Rew et al., 2021 [18]	Systematic review	Criteria for the use of puberty-blocking drugs, specific drugs used, risks, adverse consequences, and/or positive outcomes associated with their use.	Texas, USA	There were *n* = 542 participants in the nine studies included. A total of 71% of the participants in these studies required a diagnosis of gender dysphoria.	- The positive outcomes included a reduction in suicidal tendencies in adulthood, improved affect, psychological functioning, and social life. - The adverse factors included changes in body composition, slow growth, reduced height velocity, decreased bone turnover, high medication costs, and lack of insurance coverage.
Johanna Olson-Kennedy et al., 2019 [19]	Longitudinal observational study	To provide evidence-based data to inform clinical care for transgender youth and evaluate the impact of gonadotropin-releasing hormone agonists (GnRHas) administered for puberty suppression on mental health, psychological well-being, and metabolic and physiological parameters, including bone health, in a cohort of children and adolescents (Tanner stages 2–4) with gender dysphoria, comparing baseline and follow-up assessments.	USA	A total of 90 participants were enrolled in the blocker cohort and 301 in the gender-affirming hormone (GAH) cohort.	- This longitudinal and observational study collected critical data on existing models of care for transgender youth used in clinical settings for nearly a decade, despite limited empirical research supporting them.
Maria A. T. C. Van der Loos et al., 2003 [7]	Retrospective cohort observational study	To study trends in the trajectories of children and adolescents referred for gender dysphoria evaluation and/or treated following the Dutch Protocol.	Netherlands	This study was based on a retrospective cohort of 1766 children and adolescents from the Amsterdam Gender Dysphoria Cohort.	- The age of intake and initiation of GnRHa has increased over time. - The stage of puberty at GnRHa initiation has fluctuated over time. - The absence of a gender dysphoria diagnosis was the main reason for not initiating GnRHa. - Of the adolescents potentially eligible, nearly half of those who had their first visit before the age of 10 started GnRHas, compared to around two-thirds of those whose first visit occurred at age 10 or later. - The initiation of medical treatment and the need for surgical procedures depend not only on personal characteristics but also on social and legal factors.
Maureen D. Connolly et al., 2016 [9]	Systematic review	To update knowledge on the growing body of research related to the health of transgender youth that has emerged since the 2011 Institute of Medicine report on the health of lesbian, gay, bisexual, and transgender people.	USA	Fifteen articles were included, presenting quantitative data on the prevalence of transgender youth and their mental health.	- The studies included verified that transgender youth have higher rates of depression, suicidal tendencies, self-harm, and eating disorders. - The data support the importance of medical therapies and provide information on the mental health of transgender children who receive support during social transition.
Andrea L. Nos et al., 2022 [23]	Retrospective cohort study of administrative records collected between 2009 and 2018	To determine whether the use of GnRHas among transgender adolescents is associated with an increased subsequent use of growth hormones (GHs).	USA	There were a total of *n* = 434 participants, based on administrative records collected between 2009 and 2018; the analysis was completed in 2022, with a mean age (SD) of 15.4 ± 1.6 years. The participants were included in the TRICARE system of the U.S. military health plan.	- No significant association was found between the use of GnRH agonists and the later initiation of gender-affirming hormones (GAHs). - Socioeconomic status was not associated with the use of GnRHa or GH. - These findings suggest that clinicians can offer GnRH agonists to transgender and gender-diverse adolescents during pubertal development for cosmetic and mental health benefits without an increased likelihood of later use of gender-affirming hormones.
G. Gionanardi et al., 2019 [6]	Observational descriptive study	To explore the psychological aspects of hormone treatments for gender-nonconforming adults, including the controversial use of puberty suppression treatments.	Italy	There were *n* = 10 participants. They were transgender women (mean age: 37.4 years) exploring their personal histories regarding the onset and development of gender dysphoria, their experiences with hormone therapy, and their opinions on the use of GnRH analogs for puberty suppression in transgender children and adolescents.	- This study gave voice to an underrepresented group regarding the use of GnRH analogs to suppress puberty in transgender individuals, gathering firsthand information on this controversial treatment and its recommendations in international professional guidelines. - Participants valued GnRH analog treatment as an opportunity to prevent the severe body dysphoria and social phobia experienced by transgender individuals during puberty.

## Data Availability

The data presented in this study are available upon request from the corresponding author but can also be retrieved using databases.

## Public Involvement Statement

There was no public involvement in any aspect of this research.

## Guidelines and Standards Statement

This manuscript was drafted in line with the PRISMA statement.
